# Sweet Potato Leaf Feeding Decreases Cholesterol, Oxidative Stress and Thrombosis Formation in Syrian Hamsters with a High-Cholesterol Diet

**DOI:** 10.3390/life11080802

**Published:** 2021-08-09

**Authors:** Hao-Hsiang Chang, Yi-Chan Lan, Shiu-Dong Chung, Chiang-Ting Chien

**Affiliations:** 1 Department of Family Medicine, National Taiwan University Hospital, Taipei 10016, Taiwan; allanchanghs@gmail.com; 2 Department of Family Medicine, National Taiwan University College of Medicine, Taipei 10016, Taiwan; 3 Department of Family Medicine, National Taiwan University Hospital Hsin-Chu Branch, Hsinchu 30060, Taiwan; benblue56@gmail.com; 4 Department of Surgery, Division of Urology, Far-Eastern Memorial Hospital, New Taipei City 220, Taiwan; 5 Graduate Program in Biomedical Informatics, College of Informatics, Yuan-Ze University, Taoyuan City 32003, Taiwan

**Keywords:** hyperlipidemia, reactive oxygen species, sweet potato leaf, thrombosis

## Abstract

Nutritional strategies to reduce hyperlipidemia and the risk of cardiovascular disease are gaining more public favor and medical professionals’ attention. The authors of this study explored the effect of sweet potato leaf powder (SPLP) feeding on the parameters of plasma lipids, reactive oxygen species, and time to thrombosis formation in Syrian hamsters fed with high-cholesterol diets. The animals were separated into six groups: a feeding control diet, a control diet containing 0.1% cholesterol, a control diet containing 0.2% cholesterol, a control diet containing 0.1% cholesterol plus 2.5% SPLP, a control diet containing 0.1% cholesterol plus 5% SPLP, and a control diet containing 0.2% cholesterol plus 5% SPLP for six weeks. The levels of serum total cholesterol (51% increase), low-density lipoprotein cholesterol (70.6% increase), very-low-density lipoprotein cholesterol (51.3% increase), and the triglyceride and atherogenic index (LDL-C/HDL-C) significantly increased in the high-cholesterol diet groups. Concomitant 5% sweet potato leaf powder ingestion significantly decreased the lipid profiles, with a 20.6% total cholesterol reduction in the 0.1% cholesterol diet groups, a 17.2% reduction in the 0.2% group, a 48.7% LDL reduction in the 0.1% cholesterol group, and a 30.3% reduction in the 0.2% group, with a consequent decrease in the atherogenic index. SPLP feeding was found to be associated with increased fecal sterol contents, with a 188.6% increase in the 0.1% cholesterol-fed group and a 177.3% increase in the 0.2% group. The SPLP-fed groups had depressed ROS levels, elongated FeCl_3_-induced times to thrombosis formation, and increased liver superoxide dismutase contents and SREBP-1 protein expression. Sweet potato leaf intake could reduce plasma total cholesterol, LDL, and oxidative stress. We suggest sweet potato leaf intake as a choice of nutritional strategy for hyperlipidemia and cardiovascular disease prevention.

## 1. Introduction

Sweet potato, *Ipomoea batatas* *Lam.*, belongs to the Convolvulaceae family and is an important economic plant in Asia. Sweet potato root intake has been shown to reduce insulin resistance, decrease hyperlipidemia and hyperglycemia in rats [1,2], scavenge reactive oxygen species (ROS) [3,4], and protect from varied liver injuries [5,6,7]. That sweet potato root extract contains polyphenols such as anthocyanins, ferulic, and ρ-hydroxybenzoic acid probably contributes to its effects. Apart from the roots, sweet potato leaf (SPL) has become more and more popular as a vegetable in some areas for its potential health benefits. SPL has been reported to possess an antioxidant capability via the ingredients of anthocyanins, flavonoids, chlorogenic acid, ferulic, and ρ-hydroxybenzoic acid [8,9]. Some studies have shown that SPLs exert anti-mutation activity, exert anti-microbial activity, and inhibit the angiotensin-I-converting enzyme. Along with polyphenols, which can prevent cardiovascular diseases, scavenge ROS [10], and prevent tumor formation [11], dietary fibers from plants accelerate toxic material removal, and increase the ability for DNA synthesis and repair activity, thus decreasing cell injury [12,13]. SPLs also contain dietary fibers that may confer the protection effect. Other studies have shown that SPLs with specific ingredients exert anti-diabetic, anti-inflammatory, and anti-adipogenic effects, as well as promote adipocyte apoptosis in the adipocytes [14,15,16]. These data suggest the beneficial potential of SPL in the prevention of cardiovascular disease.

Hyperlipidemia and lipid peroxidation play important roles in aging, cancer risk, and cardiovascular diseases [17,18]. Excess ROS production contributes to various diseases, especially cardiovascular diseases [19,20]. In human plasma, low-density lipoprotein (LDL) is oxidized by ROS into oxLDL, a strong risk factor for inflammation and atherosclerosis [21,22]. A lipid-lowering strategy provides effective protection in normalizing vascular function and decreasing the risk of clinical events associated with atherosclerosis, and it can be combined with the ability of antioxidants to alleviate vasomotor disturbances in hypercholesterolemia and to slow the progression of atherosclerosis. There has been an increase in interest in finding a naturally antioxidant plant-based compound that could reduce increased lipid levels in plasma. In order to further our understanding of the potential of cardiovascular protection, we aimed to explore the dosage effect of SPL powder (SPLP) feeding on the lipid profiles, oxidative stress, and thrombosis formation in hamsters with 0.1% and 0.2% high-cholesterol diets. We also aimed to determine whether higher SPLP intake could increase antioxidant defense mechanisms against high-cholesterol-diet-induced oxidative stress in the animals.

## 2. Methods and Materials

### 2.1. Preparation of SPLP

Fresh sweet potato leaves (SPLs) were washed and dried at 40 ± 1 °C. After drying, the leaves were ground into powders across a 20 mesh. These powders were mixed into rat chow, as described in Table 1. We used three-week-old male Syrian hamsters purchased from the National Experimental Animal Center. SPLs were obtained from Tainung 57 of Sin-Hua City, Tainan.

### 2.2. Chemicals

Urethane, heparin, FeCl_3_, and luminol (5-amino-2, 3-dihydro-1, 4-phthalazinedione) were purchased from Sigma (St. Louis, MO, USA). The corn starch and α-cellulose were purchased from First Chemical Manufacture Co., Ltd., Taipei. Cholesterol and casein were purchased from Sigma Co. The AIN-76 mineral mixture and the AIN-76 vitamin mixture were purchased from ICN Biochemicals Co. Soybean oil was purchased from Tung-Yi Company. Pig oil was purchased from Ya-Sun Pork Company, Taipei.

### 2.3. Animals

Three-week-old male Golden Syrian hamsters (*Mesocricetus auratus*) were housed at the Experimental Animal Center, National Taiwan University with a Lab Diet (5001 Rodent diet, Harwood, USA) for one week. To explore the effects of different dosages of SPLP on high-cholesterol-diet-induced oxidative stress, the animals were divided into six groups: feeding control diet (group A), a control diet supplemented with 0.1% cholesterol (group B), a control diet supplemented with 0.2% cholesterol (group C), a control diet supplemented 0.1% cholesterol plus 2.5% SPLP (group D), a control diet supplemented 0.1% cholesterol plus 5% SPLP (group E), and a control diet supplemented with 0.2% cholesterol plus 5% SPLP (group F) for six weeks. Each group included 12 animals (four/cage), which were kept in controlled conditions at a temperature of 22 ± 2 °C, a relative humidity of 60–80%, and a 12/12 h light/dark cycle (light from 8.00 a.m. to 8.00 p.m.). Food and water were provided ad libitum. The animals with identification marks were acclimatized for 7 days before experiments. All surgical and experimental procedures were approved by the Association for Assessment and Accreditation of Laboratory Animal Care Standards in National Taiwan Normal University (No 105018) and were in accordance with the guidelines of the National Science Council of the Republic of China.

### 2.4. Collection of Blood Samples and Biochemical Analysis from Plasma

The collection of blood from six groups of animals was made on day 42 after 12 h of fasting. Hamsters were anesthetized by urethane. Blood was drawn from the jugular vein and collected in EDTA-coated tubes (3 mg/mL) by centrifugation at 1500× *g* for 5 min at 4 °C for the estimation of plasma total cholesterol and triglycerides, and high-density lipoprotein (HDL), LDL, very-low-density lipoprotein (VLDL), and cholesterol values were measured. The analysis of plasma biochemical parameters was performed with an auto-analyzer of Beckman Coulter, the ‘Synchron-CX-5 Clinical System’, by standard enzymatic methods, and assay kits were purchased from Beckman Coulter International.

### 2.5. Luminol-Enhanced Chemiluminescence Counts

We evaluated the effect of SPLP ingestion on blood ROS activity with a chemiluminescence (CL) analyzing system (CLD-110, Tohoku Electronic Industrial, Sendai, Japan), as described previously [19]. The system contains a photon detector (Model CLD-110), chemiluminescence counter (Model CLC-10), water circulator (Model CH-200), and personal computer system. A cooler circulator is connected to the Model CLD-110 photon detector to keep the temperature at 5 °C. The CLD-110 model is sensitive enough to detect as little as 10^−15^ W of radiant energy. The chemiluminescence was measured in an absolutely dark chamber of the CL analyzing system. We demonstrated that using the CL-emitting substance luminol (5-amino-2,3-dihydro-1,4-phthalazinedione, Sigma, St. Louis, MO, USA) for H_2_O_2_ or HOCl (hydrogen peroxide, sodium hypochlorite, Sigma, St. Louis, MO, USA) to enhance the CL counts provided similar data to those reported in our previous study [19]. The CL in the tested sample was continuously measured for a total of 600 s. The assay was performed in duplicate for each sample, and the results are expressed as CL counts (10 s)^−1^. The total amount of CL in 600 s was calculated by integrating the area under the curve. The mean ± SD CL level for each sample was calculated.

### 2.6. FeCl_3_-Induced Acute Arterial Thrombosis

The technique of FeCl_3_-induced acute arterial thrombosis was established in our laboratory and described previously [20]. The animals were anesthetized through the subcutaneous injection of 1.2 g/kg urethane (Sigma-Aldrich Inc., St. Louis, MO, USA). After arterial isolation, transonic flow probes (Probe# 0.5VBB517, Transonic Systems Inc., Ithaca, NY, USA) for carotid arterial blood flow measurement were applied and were displayed on a small animal blood flow meter (Model 206, Transonic Systems Inc., Ithaca, NY, USA). The blood flow signals were continuously recorded by an ADI system (PowerLab/16S, ADI Instruments, Pty Ltd., Castle Hill, Australia). The carotid artery was injured as previously described. Filter paper (1 × 2 mm) soaked with a 30% FeCl_3_ solution (ferric chloride, Sigma, St. Louis, MO, USA) was applied to the artery for 3 min, and the cavity was immediately filled with saline. The FeCl_3_ solution easily diffused into the arterial tissue, induced a Fenton-reaction-mediated endothelial injury, and subsequently induced vascular constriction to reduce arterial blood flow within minutes. The flow rate was monitored and recorded, and the time to occlusion (TTO; arterial blood flow decreases to zero) was determined.

### 2.7. Western Blot

We determined the effect of SPLP ingestion on hepatic antioxidant enzyme expression. We used 0.5 g of liver to discern the possible molecular mechanism after SPLP ingestion with Western blotting. Briefly, the homogenization, extraction, and quantification of hepatic proteins were performed. The primary antibodies raised against sheep anti-CuZnSOD (superoxide dismutase, CuZn, Oxis Health products, Inc.), sheep anti-MnSOD (superoxide dismutase, Mn: Oxis Health products, Inc.), anti-human erythrocyte catalase (Assay Designs, Inc.), sterol regulatory element-binding protein-1 (SREBP-1, Santa Cruz Biotechnology, Inc.), and monoclonal anti-β-actin (Sigma-Aldrich, Inc.) were used. Proteins on SDS-PAGE gels were transferred to nitrocellulose filters and stained. The density of the band with the appropriate molecular mass was semi-quantitatively determined with densitometry by using an image analysis system (Alpha Innotech, San Leandro, CA, USA).

### 2.8. Plasma Lipoprotein Isolation and Cholesterol Amount Assay

The blood samples were placed into sodium bromide (NaBr) solutions of different concentrations and were centrifuged with an ultracentrifuge (Beckman TL-100) at 80,000 rpm/ 4 °C for four hours (Folch, Lees, and Sloane Stanley, 1957). A density of <1.006 g/mL was identified as VLDL, 1.006–1.063 g/mL was identified as LDL, and 1.063–1.21 g/mL was recognized as HDL. We used an enzymatic endpoint method to detect the pale red color of quinoneimine with a spectrometer at 500 nm. We used the GPO-PAP method to determine the level of triglycerides.

### 2.9. Hepatic Cholesterol and Triglyceride Measurement

To extract the liver’s total cholesterol and triglyceride, 100 mg of the frozen liver tissue was dissolved in 2 mL of chloroform–methanol solution (2:1 ratio). After a complete homogenization process, the above-mentioned mixture was shaken at room temperature on a rocker for 1 h. Then, the solution was centrifuged for 20 min, and 1 mL of the supernatant (the extracted lipids) was taken and mixed with 1 mL of a Triton X-100 solution (1% in chloroform). Then, the combined solution was dried under a stream of nitrogen. To each tube of the dried lipids, 0.5 mL of deionized water was added, and the tube was placed in a water bath at 37 °C for 15 min. Afterwards, the triglyceride and cholesterol levels of the solutions were measured with a colorimetric–enzymatic method using commercial kits (Stanbio Laboratory, Boerne, TX, USA).

### 2.10. Fecal Cholesterol Analysis

We collected the feces three days before the animals were sacrificed, carefully removed hair and other materials, and then oven-dried and ground them into powders. The powders were analyzed after performing cholesterol extraction using hexane. The residue was dissolved in 5 mL of hexane, transferred to a screw top flask, dried under nitrogen, diluted with 1 mL of mobile phase, filtered through a 22 µm membrane (Millipore, Burlington, MA, USA), and injected into the HPLC system. For HPLC, we used Waters liquid chromatography (Waters, Milford, MA, USA) equipped with on-line PDA (Waters 2998) and refractive index (RID-Waters, 2414) detectors, a Rheodyne injector with a 20 µL loop, a tertiary solvent delivery system (Waters 600), an oven-heated column at 32 ± 1 °C (CTO-3840), and Empower 2 software. The analytical column was a 300 × 3.9 × 4 mm Nova Pack CN HP (Waters, Milford, MA, USA). The mobile-phase was n-hexane:2-propanol (97:3, *v*/*v*) at a flow rate of 1 mL/min and an analysis time of 30 min. The HPLC solvents were filtered through a 22 mm Millipore membrane (Bedford, MA, USA) under vacuum prior to use. Quantification was conducted by external standardization, with a concentration ranging from 0.1 to 1.8 mg/mL.

### 2.11. Statistical Analyses

All values were expressed as mean ± standard deviation (SD). Differences within groups were evaluated by paired *t*-tests. The data were statistically analyzed by one-way analysis of variance, followed by Tukey’s multiple comparison tests. Differences were regarded as significant if *p* < 0.05 was adapted. Statistical analyses were performed using SPSS 18.0 (IBM, Armonk, NY, USA), and curve fitting was carried out using GraphPad Prism (v. 6.0).

## 3. Results

The composition of the ingested food in the six groups of animals is shown in Table 1. The caloric density of all the formula diets was 3.3 kcal/g, which conferred 21.7–24.75 kcal a day (3.3 kcal × 6.6–7.5 g/day). All hamsters received these diets indiscriminately. There was no difference in the body weight gain, food intake, and food efficiency (increased body gain weight/food intake) among these six groups, as shown in Table 2. This means that the different formulae of the food preparation did not affect the growth and development of these hamsters.

The high-cholesterol diet in groups B and C induced significantly higher total cholesterol, VLDL, and LDL levels than the control diet. The highest total cholesterol level was noted in group C with the 0.2% cholesterol diet, and it was 34% higher than that in control diet group A and 24% higher than that in 0.1% cholesterol diet group B. Feeding with a diet containing 2.5% (group D) or 5% SPLP (group E) significantly decreased the plasma total cholesterol level by 15.5% and 20.6%, respectively, compared to that of cholesterol diet group B (0.1% cholesterol). In addition, 5% SPLP diet ingestion significantly reduced the plasma cholesterol value by 17.4% in group F compared to group C with the 0.2% cholesterol diet. Similar trends were noted regarding the LDL level; the cholesterol-containing diet increased the LDL level by 44.9% in group B (0.1% cholesterol) and 70.6% in group C (0.2% cholesterol). SPLP feeding reduced the LDL level in the 0.1% cholesterol-fed groups by 29.5% compared to group D (2.5% SPLP) and 48.7% compared to group E (5% SPLP). The higher SPLP-containing diet (5% SPLP) significantly reduced LDL (48.7%) in the 0.2 cholesterol-fed group. The cholesterol-containing diet did not affect the HDL levels. SPLP feeding seemed to increase HDL levels, but no statistically significant differences were observed among the six groups. Our results also showed that the atherogenic index increased with cholesterol feeding. A 60% increase was observed in the 0.1% cholesterol-fed group, and a 68% increase was observed in the 0.2% cholesterol group. Both 2.5% and 5% SPLP ingestion significantly reduced the atherogenic index, as shown in Table 3. As for VLDL-C, the ingestion of the 0.1% or 0.2% cholesterol diet significantly increased the VLDL-C level in groups B and C. The ingestion of 2.5% SPLP in group D or 5% SPLP in group E significantly depressed the VLDL-C level by 63.4% or 62%, respectively, compared to that in the relevant control groups. The plasma triglyceride levels were not significantly changed by either the high-cholesterol diet or SPLP feeding.

Table 4. illustrates the hepatic cholesterol, triglyceride, and fecal cholesterol levels among animal groups. Hepatic cholesterol levels were significantly elevated in the 0.1% and 0.2% cholesterol-containing diet groups. Concomitant SPLP feeding reduced hepatic cholesterol by 29.0% in the 2.5% SPLP group and 32.0% in the 5% SPLP group compared to the relevant control groups (0.1% cholesterol), and there was a 33.9% decrease for the 5% SPLP diet in the 0.2% cholesterol group. There was no difference in hepatic triglyceride among the six groups (*p* > 0.05). The fecal cholesterol amount increased by 40.25% and 65.35% in the 0.1% and 0.2% cholesterol diet control groups, respectively. SPLP feeding significantly increased the fecal cholesterol amount by 67.3%, 188.6%, and 177.3% in groups D, E, and F, respectively.

Table 5 shows the carotid artery blood flow, arterial blood pressure, time to occlusion, and index of ROS among animal groups. The baseline levels of carotid blood flow and pressure were not different in the six groups. The time for FeCl_3_-induced arterial occlusion (TTO) was elongated by 4.1- and 1.19-fold compared to group B in SPLP feeding groups D and E. TTO was elongated by 3.65-fold in group F compared to group C. The ROS index and blood luminol increased in the cholesterol diet groups, with a 31.2% increase in the 0.1% group and a 149.3% increase in the 0.2% group. SPLP ingestion decreased blood luminol, with 59.7% and 89.0% reductions in groups D (2.5% SPLP) and E (5% SPLP) compared to group B (0.1% cholesterol). In addition, a remarkable reduction of 93.2% compared to the 0.2% cholesterol control group was observed in the 5% SPLP ingestion group.

**Figure 1:**
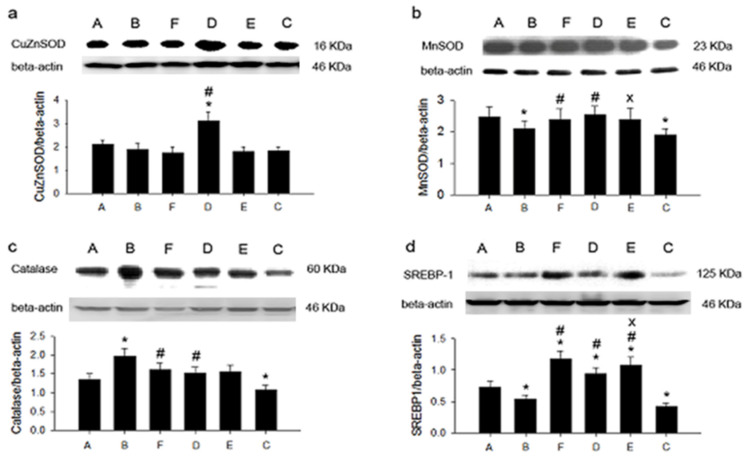
The expression of hepatic antioxidant proteins and SREBP-1 in six groups. (**a**): CuZnSOD; (**b**): MnSOD; (**c**): catalase; (**d**): SREBP-1. * *p* < 0.05 vs. group C. # *p* < 0.05 vs. group E. x *p* < 0.05 vs. group F. C: control diet; E: control diet plus 0.1% cholesterol; A: control diet plus 0.1% cholesterol plus 2.5% sweet potato leaf powder; B: control diet plus 0.1% cholesterol plus 5% sweet potato leaf powder; D: control diet plus 0.2% cholesterol plus 5% sweet potato leaf powder; F: control diet plus 0.2% cholesterol.

Figure 1 illustrates hepatic protein expression, including CuZnSOD, MnSOD, catalase, and SREBP-1, among animal groups. The highest expression of CuZnSOD was found in group B with the 0.1% cholesterol diet plus 5% SPLP. MnSOD expression was also highest in group B, indicating that 5% SPLP ingestion enhanced CuZnSOD and MnSOD expression. Catalase expression was also greatly enhanced in group B. The level of SREBP-1 expression was consistently elevated in groups B and D. SPLP enhanced hepatic CuZnSOD, MnSOD, catalase, and SREBP-1 expression.

## 4. Discussion

According to global and regional data, the largest numbers of deaths from non-communicable diseases are estimated to be caused by cardiovascular diseases [23]. The central pathophysiology of cardiovascular disease is the formation of atherosclerosis, in which LDL plays a key role. One of the most effective ways to reduce CVD risk is to reduce LDL level [18]. Statins have been proven to have beneficial effects on CVD reduction and are recommended by clinical guidelines [24,25,26]. However, the adverse effects of statins, such as muscle aches and elevated liver enzyme levels, limit the application of statins for those who are not tolerant [27]. Additionally, dietary intervention with low cholesterol has little effect on lowering blood cholesterol [28]. Natural food or supplements with lipid-lowering effects are expected to fit the gap. The present study has demonstrated that the dietary intake of sweet potato leaves has the potential to decrease plasma total cholesterol and LDL levels, probably through a decrease in cholesterol absorption and the inhibition of hepatic cholesterol synthesis. The findings of this study provide an alternative or complementary strategy to hypercholesterolemia management and cardiovascular disease prevention.

SPLs are traditional foods for treating cardiovascular disease in Africa [29]. According to our data, the ingestion of SPLP exerts a moderate reduction in plasma cholesterol and LDL levels in animals fed with a high-cholesterol diet. In addition, such a cholesterol-lowering effect works in a dose-responsive manner. A diet containing 5% SPL may exert a 20% reduction in plasma LDL, which may contribute to an estimated 20% cardiovascular risk reduction [30]. The mechanisms of SPL’s cholesterol-lowering effect were also investigated in this study. We explored the fecal cholesterol amount and lipid homeostasis in the liver, which represent the two main resources of plasma cholesterol: absorption from the gut and synthesis in the liver. The fecal-cholesterol-expelling amount increased in the SPLP-fed groups in this study, an increase that is crucial to the cholesterol-lowering effect of a high-cholesterol diet.

The ingredients of anthocyanins, flavonoids, chlorogenic acid, ferulic, and ρ-hydroxybenzoic acid provide SPL with its antioxidant capability [8,9]. Additionally, SPLs containing dietary fibers may exert anti-diabetic, anti-inflammatory, and anti-adipogenic effects, as well as promote adipocyte apoptosis in adipocytes [14,15,16]. In the present study, in addition to the LDL-lowering effect, SPLP ingestion also evidenced potential to reduce oxidative stress. Our data, obtained with an ultrasensitive chemiluminescence analyzer, show that the blood ROS was higher in hamsters with high-cholesterol diets. When concomitantly feeding with SPLP, the blood ROS indexes were significantly reduced from 1224 to 83 counts/10 s. This decrease in ROS may have arisen from direct and indirect ROS-scavenging mechanisms. The data also indicate that Tainung 57 contained the highest total polyphenol (2839 mg of GAE (total phenolics expressed as gallic acid equivalents) per 100 g of SPL) and flavonoid content (607.6 mg QE (flavonoid contents expressed as quercetin equivalents) per 100 g of SPL) [9,31]. Since it is rich in antioxidants, SPL has direct ROS-scavenging potential. Western blotting analysis showed that hepatic CuZnSOD, MnSOD, and catalase expression were higher in the SPLP-fed group with the 0.2% high-cholesterol diet. SPL effectively reduced blood ROS through the antioxidant action of abundant polyphenols and increased the amount of intrinsic scavengers. We suggest that SPLP intake increased the polyphenol and dietary fiber absorption, thus leading to a decreased ROS level, increased antioxidant enzyme expression, and increased cholesterol synthesis inhibition.

Excess ROS leads to the increased oxidation of LDL. Oxidized LDL (oxLDL) has been proven to play a key role in atherosclerosis and thrombosis [32]. oxLDL tends to evoke an inflammatory response and promotes thrombotic formation [19,32]. SPL ingestion reduces ROS and results in decreased oxLDL levels, which was noted in a previous study [33]. Our study, which used an FeCl_3_-induced thrombosis model, found that SPLP ingestion elongated the occlusion time. The results suggest that SPLP may ameliorate thrombosis formation through reduced ROS and oxLDL levels. The use of intravenous hydrogen-rich saline [34], orally ingested dietary fiber^20^, hot bathing via progressive thermal preconditioning [35], and SPLP oral intake in the present study conferred antioxidant protection and improved atherosclerosis formation. Based on this information, enhanced endogenous or exogenous antioxidant defense mechanisms such as SPL intake may be a preventive or therapeutic target to ameliorate cardiovascular diseases.

SPL has been noted for its beneficial effects in the treatment of hyperglycemia or as a food additive for the prevention of type 2 diabetes via the activation of GLUT4 and the regulation of the PI3K/AKT pathway [36]. In addition, an extract of sweet potato exerts hepatic protection and treatment potential for non-alcoholic fatty liver disease [6,37]. In our study, hepatic SREBP-1 expression was elevated by SPLP feeding. SREBP-1c mainly activates genes encoding enzymes required in fatty acid and triglyceride biosynthesis, while SREBP-1a regulates genes involved in both cholesterol and fatty acid biosynthesis. SPL also shows mechanistic potential for liver protection from non-alcoholic fatty liver disease.

This study had some limitations. In an animal model, hypercholesterolemia is caused by a high-cholesterol diet, while genetic predisposition is the main etiology of hypercholesterolemia in humans. The application of our results to general human populations mandates further evaluation. Another concern is the proper intake amount of SPL to achieve the cholesterol-lowering effect for humans, which mandates further study.

SPL is becoming more and more popular as a vegetable and functional food, and it possesses several health benefits, including anti-diabetic, anti-cancer, and anti-inflammatory effects. Our results demonstrate that SPL can decrease the levels of total plasma cholesterol, LDL, and the atherogenic index, possibly through its antioxidant ingredients or dietary fibers that increase fecal cholesterol excretion. Additionally, SPL may depress plasma ROS by increasing antioxidant enzyme expression, including that of CuZnSOD, MnSOD, catalase, and its abundant antioxidants. SPL or its supplement may provide a choice of nutritional therapy to prevent and reduce cardiovascular events.

## Figures and Tables

**Table 1:** Composition of the test diets in these six groups.

Group Ingredients	A	B	C	D	E	F
Casein	20%	20%	20%	19.50%	19.00%	19.00%
Corn	37.25%	37.15%	37.05%	36.22%	35.29%	35.29%
Starch	20%	20%	20%	19.50%	19.00%	19.00%
Sucrose	6.84%	6.84%	6.84%	6.67%	6.50%	6.50%
Pig oil	3.16%	3.16%	3.16%	3.08%	3.00%	3.00%
Bean oil	1.25%	1.25%	1.25%	1.22%	1.19%	1.19%
Vitamins	3.5%	3.5%	3.5%	3.41%	3.33%	3.33%
Minerals	5%	5%	5%	4.88%	4.75%	4.75%
Cellulose	3%	3%	3%	2.93%	2.85%	2.85%
Cholesterol	0%	0.1%	0.2%	0.1%	0.1%	0.2%
SPLP	0%	0%	0%	2.5%	5%	5%

A: control diet; B: control diet plus 0.1% cholesterol; C: control diet plus 0.2% cholesterol; D: control diet plus 0.1% cholesterol plus 2.5% sweet potato leaf powder; E: control diet plus 0.1% cholesterol plus 5% sweet potato leaf powder; F: control diet plus 0.2% cholesterol plus 5% sweet potato leaf powder.

**Table 2:** Weight gain and feed efficiency of the male hamster.

	Weight Gain (gm)	Feed Intake (gm)	Feed Efficiency ** (%)
A	36 ± 4	322 ± 30	11 ± 1
B	31 ± 5	301 ± 29	10 ± 1
C	38 ± 6	354 ± 38	10 ± 1
D	25 ± 5	281 ± 27	10 ± 1
E	30 ± 4	307 ± 34	10 ± 1
F	30 ± 3	330 ± 35	9 ± 1

A: control diet; B: control diet plus 0.1% cholesterol; C: control diet plus 0.2% cholesterol; D: control diet plus 0.1% cholesterol plus 2.5% sweet potato leaf powder; E: control diet plus 0.1% cholesterol plus 5% sweet potato leaf powder; F: control diet plus 0.2% cholesterol plus 5% sweet potato leaf powder. ** Feed efficiency = (weight gain/feed intake) × 100. Mean ± SD are shown in the same column for values not sharing a common superscription. A one-way analysis of variance was used to establish differences among groups.

**Table 3:** The plasma cholesterol profiles and atherogenic index (AI) in these six groups of hamsters.

	Total-C	VLDL-C	LDL-C	HDL-C	AI
A	85.88 ± 0.97 ^d^	12.36 ± 2.37 ^c^	19.02 ± 2.03 ^bc^	47.25 ± 5.04 ^a^	0.40 ± 0.08 ^b^
B	112.79 ± 10.46 ^b^	31.11 ± 15.22 ^a^	27.56 ± 54.21 ^bc^	42.75 ± 17.40 ^a^	0.64 ± 0.24 ^ab^
C	130.82 ± 5.18 ^a^	18.70 ± 2.62 ^bc^	32.46 ± 3.74 ^a^	47.11 ± 6.12 ^a^	0.69 ± 0.09 ^a^
D	95.30 ± 8.91 ^c^	11.36 ± 4.14 ^c^	19.44 ± 5.99 ^c^	53.15 ± 4.72 ^a^	0.36 ± 0.07 ^b^
E	89.55 ± 2.37 ^cd^	11.82 ± 6.25 ^c^	14.14 ± 3.26 ^bc^	55.02 ± 7.89 ^a^	0.25 ± 0.12 ^ab^
F	108.29 ± 8.59 ^b^	23.99 ± 10.99 ^ab^	22.61 ± 8.16 ^b^	54.82 ± 10.71 ^a^	0.41 ± 0.18 ^ab^

A: control diet; B: control diet plus 0.1% cholesterol; C: control diet plus 0.2% cholesterol; D: control diet plus 0.1% cholesterol plus 2.5% sweet potato leaf powder; E: control diet plus 0.1% cholesterol plus 5% sweet potato leaf powder; F: control diet plus 0.2% cholesterol plus 5% sweet potato leaf powder; VLDL-C: very-low-density lipoprotein-cholesterol; LDL-C: low-density lipoprotein-cholesterol; HDL-C: high-density lipoprotein-cholesterol; AI: atherogenic index = LDL-C/HDL-C. Mean ± SD are shown in the same column for values not sharing a common superscription. An analysis of variance was used to establish differences among groups. Letters were found to be significantly different from one another with Tukey’s test (*p* < 0.05). The same superscripts small letters(a–d) were found to be not significantly different from one another with Tukey’s test.

**Table 4:** Liver cholesterol, triglyceride, and fecal cholesterol contents of the male hamster.

	Liver Total Cholesterol (mg/g)	Liver Triglycerol (mg/g)	Fecal Cholesterol (mg/day)
A	8.32 ± 1.85 ^c^	0.48 ± 0.05 ^a^	5.67 ± 0.68 ^d^
B	15.00 ± 3.04 ^bc^	0.52 ± 0.07 ^a^	6.13 ± 1.67 ^d^
C	23.01 ± 1.72 ^a^	0.53 ± 0.09 ^a^	7.61 ± 0.28 ^d^
D	10.63 ± 2.97 ^bc^	0.57 ± 0.03 ^a^	10.26 ± 1.57 ^c^
E	10.19 ± 2.17 ^bc^	0.52 ± 0.06 ^a^	17.69 ± 3.35 ^b^
F	15.22 ± 2.98 ^b^	0.49 ± 0.76 ^a^	21.10 ± 1.86 ^a^

A: control diet; B: control diet plus 0.1% cholesterol; C: control diet plus 0.2% cholesterol; D: control diet plus 0.1% cholesterol plus 2.5% sweet potato leaf powder; E: control diet plus 0.1% cholesterol plus 5% sweet potato leaf powder; F: control diet plus 0.2% cholesterol plus 5% sweet potato leaf powder. Mean ± SD are shown in the same column for values not sharing a common superscription. An analysis of variance was used to establish differences among groups. Letters were found to be significantly different from one another with Tukey’s test (*p* < 0.05). The same superscripts small letters(a–d) were found to be not significantly different from one another with Tukey’s test.

**Table 5:** The effect of sweet potato leaf on ROS level and time to occlusion in the hamsters.

	Carotid Blood Flow (mL/min)	Time to Occlusion (Second)	Luminol ROS CL (Counts/10 s)	Blood Pressure (mmHg)
A	0.84 ± 0.26 ^a^	516 ± 128 ^b^	491 ± 25 ^cb^	95 ± 8 ^a^
B	0.42 ± 0.47 ^a^	470 ± 125 ^b^	644 ± 25 ^b^	97 ± 13 ^a^
C	0.39 ± 0.25 ^a^	455 ± 101 ^b^	1224 ± 210 ^a^	103 ± 6 ^a^
D	0.45 ± 0.08 ^a^	617 ± 151 ^b^	259 ± 21 ^c^	100 ± 8 ^a^
E	0.51 ± 0.19 ^a^	1935 ± 585 ^a^	71 ± 8 ^d^	104 ± 21 ^a^
F	0.54 ± 0.49 ^a^	2018 ± 717 ^a^	83 ± 17 ^d^	96 ± 15 ^a^

A: control diet; B: control diet plus 0.1% cholesterol; C: control diet plus 0.2% cholesterol; D: control diet plus 0.1% cholesterol plus 2.5% sweet potato leaf powder; E: control diet plus 0.1% cholesterol plus 5% sweet potato leaf powder; F: control diet plus 0.2% cholesterol plus 5% sweet potato leaf powder. Mean ± SD are shown in the same column for values not sharing a common superscription. An analysis of variance was used to establish differences among groups. Letters were found to be significantly different from one another with Tukey’s test (*p* < 0.05). The same superscripts small letters(a–d) were found to be not significantly different from one another with Tukey’s test.
